# Alkali-Stable Anion Exchange Membranes Based on Poly(xanthene)

**DOI:** 10.1021/acsmacrolett.2c00672

**Published:** 2022-12-20

**Authors:** Dong Pan, Si Chen, Patric Jannasch

**Affiliations:** Polymer & Materials Chemistry, Department of Chemistry, Lund University, P.O. BOX 124, SE-221 00 Lund, Sweden

## Abstract

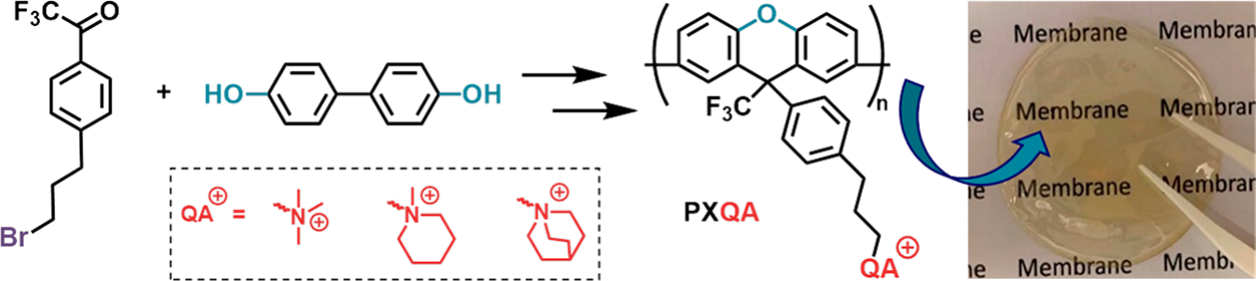

Poly(xanthene)s (PXs) carrying trimethylammonium, methylpiperidinium, and quinuclidinium cations were synthesized and studied as a new class of anion exchange membranes (AEMs). The polymers were prepared in a superacid-mediated polyhydroxyalkylation involving 4,4′-biphenol and 1-bromo-3-(trifluoroacetylphenyl)-propane, followed by quaternization reactions with the corresponding amines. The architecture with a rigid PX backbone decorated with cations via flexible alkyl spacer chains resulted in AEMs with high ionic conductivity, thermal stability and alkali-resistance. For example, hydroxide conductivities up to 129 mS cm^–1^ were reached at 80 °C, and all the AEMs showed excellent alkaline stability with less than 4% ionic loss after treatment in 2 M aq. NaOH at 90 °C during 720 h. Critically, the diaryl ether links of the PX backbone remained intact after the harsh alkaline treatment, as evidenced by both ^1^H NMR spectroscopy and thermogravimetry. Our combined findings suggest that PX AEMs are viable materials for application in alkaline fuel cells and electrolyzers.

Polymeric anion exchange membranes (AEMs) are crucial components in alkaline energy conversion and storage systems such as anion exchange membrane fuel cells (AEMFCs) and electrolyzer cells (AEMECs).^1−5^ The solid electrolyte membrane physically separates the electrodes and the feed gases while facilitating efficient OH^–^ ion transport and providing necessary mechanical support for the catalyst layers in the cell. An intensive development of AEMFCs and AEMECs as potentially more sustainable and cost-effective alternatives to the corresponding proton exchange membrane fuel cells (PEMFCs) and electrolyzer cells (PEMECs) has placed a considerable focus on synthetic approaches toward durable and high-performing AEMs.^6,7^ Especially, the alkaline stability of currently available AEMs is generally insufficient, which critically limits their life span in real applications.^8−10^ The structural integrity of the AEM is seriously challenged by the highly basic and nucleophilic OH^–^ ions, which may chemically attack the polymer backbone and especially the cations. Moreover, the OH^–^ ion has a lower mobility in dilute aqueous solution than H^+^,^11^ which generally makes it difficult for AEMs to reach the same conductivity as the corresponding proton exchange membranes.

Synthetic approaches toward highly conductive and chemically stable AEMs are currently intensively pursued via direct polymerization and postpolymerization functionalization strategies.^12^ Especially, membrane materials based on highly rigid aromatic polymers with contorted structures have received growing interest.^13−18^ In this context, various polyhydroxyalkylation procedures are now developed as attractive pathways to rigid and high molecular weight aromatic polymers which may serve as a starting point for durable and processable AEM materials.^19,20^ Polyhydroxyalkylation is a type of Friedel–Crafts polycondensation reaction where, e.g., a trifluoromethyl ketone reacts with electron-rich arene in a superacidic medium to yield a polymer with high molecular weight (>100 kDa) and narrow polydispersity.^21^ Here, the polymer structure is highly tunable because of the wide choice of monomers. By rational monomer selection and design, polymers with a tailored structure can be synthesized to significantly advance AEM properties.^19,20,22^ For example, AEMs based on poly(arylene piperidinium)s^20,23^ and poly(arylene alkylene)s^19,24^ have recently demonstrated excellent performances.

Polyxanthenes (PXs) are another type of aromatic polymers that have been prepared in polyhydroxyalkylations.^13^ The xanthene unit consists of a pyran ring fused with two benzene rings. Inclusion of this rigid planar tricyclic aromatic heterocycle in the polymer backbone may induce intrinsic microporosity which may in turn facilitate both ion and gas transport. Lately, sulfonated PX membranes have been reported to reach high proton conductivity at relatively low ion exchange capacities (IECs).^15^ Subsequently, the membranes were applied in the production of osmotic energy with a high efficiency.^17^ Moreover, nonionic PXs have been found to enable fast gas transport due to a considerable free volume fraction provided by the rigid xanthene units.^18^ Although the quite unique molecular structure of PX shows great potential for membrane applications, it has not yet been explored and evaluated for AEMs. Potentially, there is a risk that the ether bond of the xanthene unit may undergo hydrolysis in strongly alkaline environments. However, scission of the ether bond in PX will lead to opening of the pyran ring, accompanied by reduced backbone chain rigidity, but not to detrimental polymer chain cleavage that eventually leads to complete loss of mechanical integrity.

In the present work, we report on the first AEMs based on PX backbones. A polyxanthene (PX-Br) was first synthesized in a polyhydroxyalkylation–cyclodehydration sequence involving 4,4′-biphenol and a bromoalkylated trifluoroacetophenone monomer. Next, a series of three QA-functionalized PXs were prepared in Menshutkin reactions of PX-Br with trimethyl amine, methylpiperidine, and quinuclidine, respectively, to obtain the corresponding cationic polymers PXTMA, PXmPip, and PXQui (Scheme 1). Finally, AEMs were cast and characterized with respect to chemical structure, water uptake, OH^–^ conductivity, and stability, with a special focus on the alkaline stability of the xanthene ether bond.

**Scheme 1:**
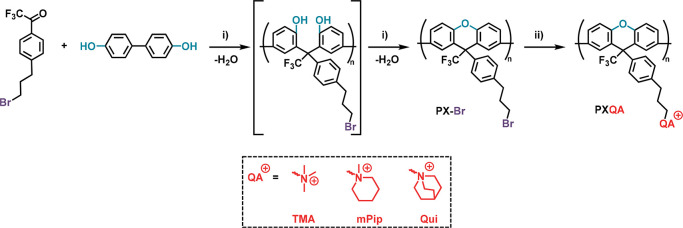
Synthetic Route to Polyxanthenes Tethered with Quaternary Ammonium Cations Key: (i) polyhydroxyalkylation and cyclodehydration: dichloromethane (DCM), triflic acid, at 25 °C; (ii) quaternization: dimethylacetamide (DMAc), trimethylamine solution at 25 °C/*N*-methylpiperidine and quinuclidine at 85 °C.

The bromoalkylated trifluoroacetophenone monomer [1-bromo-3-(trifluoroacetylphenyl)-propane, TFAp-Br] was synthesized in high yield (92%) via a one-step Friedel–Crafts trifluoroacylation of 1-bromo-3-phenylpropane using trifluoroacetic anhydride (employing a modified previously published procedure^25^). The structure of the product was confirmed by ^1^H, ^13^C, and ^19^F NMR spectroscopy (Figure S1, detailed synthetic procedure and NMR analysis are presented as Supporting Information). Notably, the acylation reaction resulted in about 94% para-substitution and 6% ortho-substitution. Next, TFAp-Br was employed in a superacid-mediated polyhydroxyalkylation with 4,4′-biphenol at 25 °C (Scheme 1). In the presence of trifluoromethanesulfonic acid (TFSA), the carbonyl group of TFAp-Br is protonated to form a highly electrophilic carboxonium ion, which reacts with the electron-rich biphenol monomer. The carbinol intermediate is then protonated and reacts with a second biphenol monomer. Subsequently, the two neighboring phenol groups undergo cyclodehydration, resulting in the formation of the xanthene unit (Scheme 1). Using a stoichiometric feed of the two monomers, the polymerization was quite efficient, and within hours a white and fibrous polymer product was obtained. Notably, the polyhydroxyalkylation reaction proceeded with high regioselectivity at the ortho-position of the hydroxyl groups.^13^ The ^1^H NMR spectrum of the polymer (PX-Br) showed signals from the xanthene rings between δ = 6.8 and 7.4 ppm (Figure S2). In addition, signals from the alkyl side chains were found at 2.24, 2.86, and 3.46 ppm, respectively, with the expected intensity ratios 1:1:1. No phenolic (Ph–OH) protons were detected and hence confirmed the complete cyclodehydration of the adjacent phenol groups to form the xanthene units. PX-Br was highly soluble in tetrahydrofuran (THF), dichloromethane (DCM), chloroform, dimethylacetamide (DMAc), and *N*-methyl-2-pyrrolidone (NMP), which further supported the absence of phenol groups. The polymer had a molecular weight of *M*_n_ = 117 kDa and a dispersity of *Đ* = 4.1, as determined by size exclusion chromatography, and formed transparent and robust films when cast from a chloroform solution. Thermogravimetric analysis (TGA) showed that PX-Br had high thermal stability with a decomposition temperature *T*_d,95_ = 361 °C (Figure S3), and differential scanning calorimetry (DSC) indicated a glass transition temperature (*T*_g_) at 280 °C (Figure S4).

The QA cations were introduced by Menshutkin reactions of PX-Br with trimethylamine, *N*-methylpiperidine, and quinuclidine, respectively (Scheme 1). ^1^H NMR analysis of the resulting cationic polymers showed signals from the methylene protons (*a–c*) of the alkyl side chains at 2.0, 2.6, and 3.3 ppm, respectively (Figure 1). In the case of PXTMA, the signal of the methyl protons (*d*) was located at 3.0 ppm. For the cyclic QAs (mPip and Qui), the α-protons (*e*) in the rings gave rise to signals around 3.3 ppm. The signals from the other methylene protons in the rings appeared between 1.2–2.2 and 1.5–2.2 ppm for PXmPip and PXQui, respectively. The signal intensity ratio of the protons of the QA groups and the xanthene backbone confirmed the complete displacement of the bromine atoms (Figure S2). In addition, Mohr titrations of the Br^–^ content gave ion exchange capacity (IEC) values in excellent agreement with the theoretical values between 2.03 and 2.26 mequiv g^–1^ (Table S1). All the cationic polymers were readily soluble in methanol, DMAc, NMP, and dimethyl sulfoxide (DMSO), implying good AEM processability.

**Figure 1:**
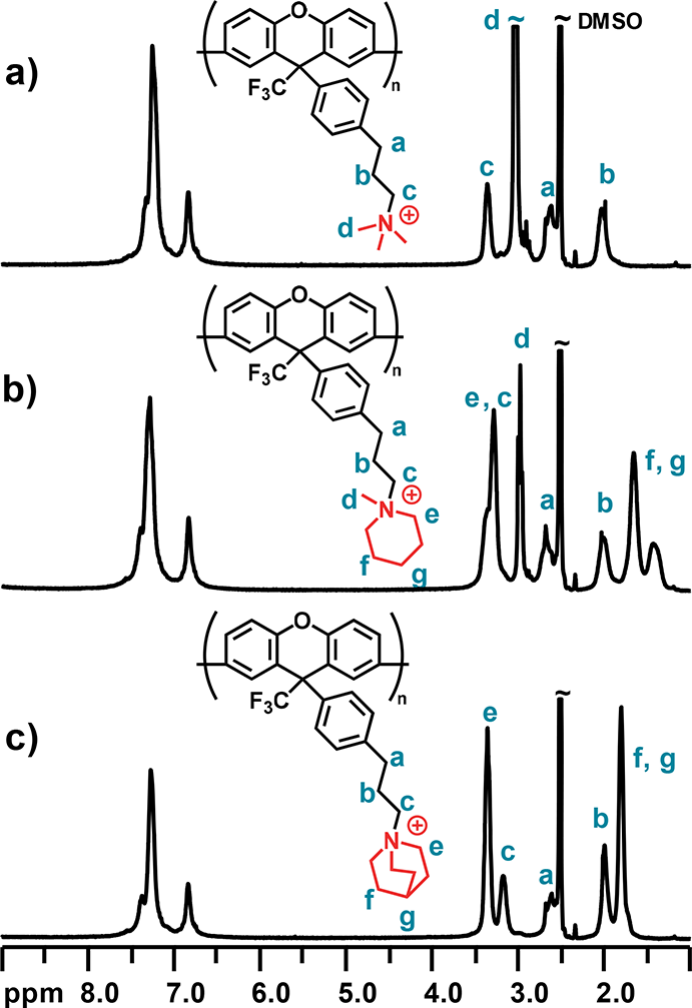
^1^H NMR spectra of (a) PXTMA, (b) PXmPip, and (c) PXQui in DMSO-*d*_6_ containing 5 vol % trifluoroacetic acid (TFA).

AEMs with a thickness of 60 μm were cast from DMSO solutions of the polymers at 80 °C. The AEMs were fully transparent and mechanically flexible (Figure S5), most probably resulting from the high molecular weight of PX-Br. Initial results revealed that when *M*_n_ was below approximately 100 kDa the resulting AEMs were free-standing but quite brittle. The AEMs exhibited high thermal stability and started to decompose only above 240–300 °C, corresponding to the loss of the QA cations (Figure S3). The Qui cation was the most thermally stable cation and gave PXQui the highest *T*_d,95_ at 303 °C (Table S2). Subsequently, the AEMs showed a second weight loss at around 500 °C that was attributed to the decomposition of the polyxanthene backbone, similar to that observed with PX-Br (Figure S3).

The morphology of the AEMs was studied by small-angle X-ray scattering (SAXS) measurements on samples in the Br^–^ form in an ambient air atmosphere. As shown in Figure S6, the samples did not show any sharp scattering peaks. Still, PXTMA displayed a rather broad ionomer peak at around *q* = 2.8 nm^–1^, which corresponded to a *d*-spacing of 2.21 nm. PXmPip and PXQui showed very weak, less discernible ionomer peaks in the same range. Presumably, the highly rigid polyxanthene backbone prevented regular ionic clustering (phase separation) even though the local ionic mobility was facilitated by the flexible alkyl spacer chains. Recently, a series of rigid polyfluorenes functionalized with the same QA cations have previously been found to exhibit similar weak ionomer peaks in the same *q* range.^26^

The water uptake (WU) of the AEMs in the OH^–^ form was measured between 20 and 80 °C, and the results were found to be only slightly influenced by the nature of the QA group. As shown in Figure 2a and Table S1, the water uptake of the AEMs increased sharply with the temperature, from 75–92% at 20 °C to 149–175% at 80 °C. PXTMA had the highest water uptake, as well as the highest in-plane and through-plane swelling ratios (Table S1 and Figure S7), due to the highest IEC. Still, all the AEMs remained intact during all the measurements. In general, the water uptake of the PX AEMs was relatively high in comparison with, e.g., poly(arylene alkylene)s at a similar ionic content (Table S3).^19,27^ For example, an AEM based on poly(*p*-terphenyl alkylene) carrying TMA cations with an IEC of 2.16 mequiv g^–1^ reportedly showed a water uptake of 65% at 80 °C, which is less than half compared to the PX AEMs.^27^ This may be a consequence of the rigid xanthene backbone as well as the phenylene unit in the tether, which inhibited dense polymer packing and promoted excess free volume for water adsorption.

**Figure 2:**
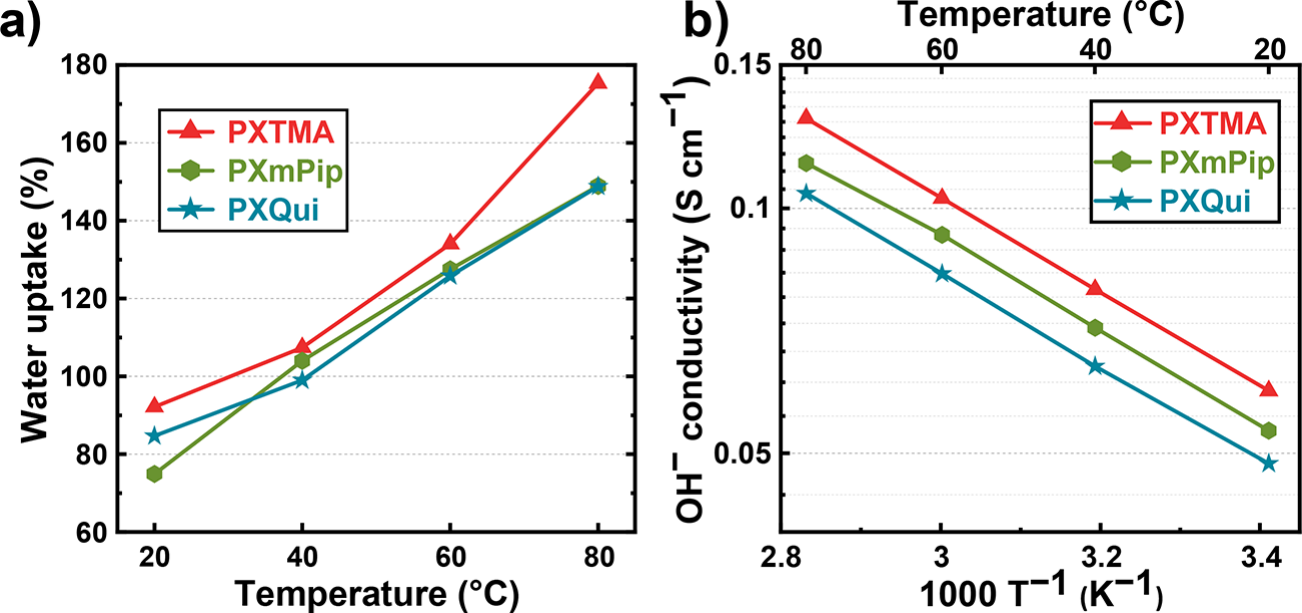
(a) Water uptake and (b) OH^–^ conductivity of the AEMs measured in the fully hydrated (immersed) state between 20 and 80 °C.

High hydroxide conductivity is an essential property of AEMs in order to support a high performance in AEMFCs and AEMECs. The hydroxide conductivity of the present AEMs was measured between 20 and 80 °C, and the results are displayed in Figure 2b and Table S1. In general, all the AEMs reached high OH^–^ conductivity, above 100 mS cm^–1^ at 80 °C. Specifically, PXTMA with the highest IEC demonstrated the highest conductivity with 60 and 129 mS cm^–1^ at 20 and 80 °C, respectively. Hence, these AEMs achieved higher conductivities than most of the reported poly(arylene alkylene) AEMs at similar IEC values (Table S3). This may be attributed to favorable levels of both the IEC and the water uptake. Moreover, the conductivities of the AEMs showed an Arrhenius behavior (Figure 2b) with apparent activation energies (*E*_a_s) around 11 kJ mol^–1^ between 20 and 80 °C, which was comparable with previously reported values for AEMs (10–14 kJ mol^–1^).^26,28,29^

The structural integrity of AEMs is challenged by the chemically aggressive OH^–^ ions, especially at elevated temperatures and alkali concentrations. To evaluate the alkaline stability of the present AEMs, samples were immersed in 1 M aq. NaOH at 80 °C, as well as in 2 M aq. NaOH at 90 °C for 720 h. ^1^H NMR spectra were then recorded by dissolution of the samples in DMSO-*d*_6_ with addition of TFA (5 vol %). TFA was added to protonate any tertiary amine groups formed as degradation products after ionic loss, giving rise to a distinct singlet above 9.0 ppm. ^1^H NMR analysis revealed no change in the molecular structure after alkali treatment under the former (milder) conditions and only a barely detectable degradation under the latter (harsher) conditions, as shown in Figure 3. In all the spectra, the shape and integral of the aromatic signals between 6.6 and 7.6 ppm remained unchanged, indicating a high alkaline stability of the polyxanthene backbone, including the diaryl ether bond. After treatment in 2 M aq. NaOH, the spectra of PXTMA and PXmPip revealed some very small emerging signals (Figure 3a,b), while the spectrum of PXQui was virtually identical with the initial one (Figure 3c). For PXTMA, a tiny singlet appeared at 9.6 ppm, which hinted at a protonated tertiary amine group resulting from methyl substitution of the TMA cation. In the spectrum of PXmPip, new small signals are seen between 4.5 and 6.2 ppm (Figure 3b). These were characteristic signals from vinylic protons resulting from Hofmann elimination, which may occur in the alkyl chain or in the piperidinium ring. Since almost no protontated amine signals were detected for this sample, it is likely that Hofmann elimination mostly occurred at the alkyl spacer.

**Figure 3:**
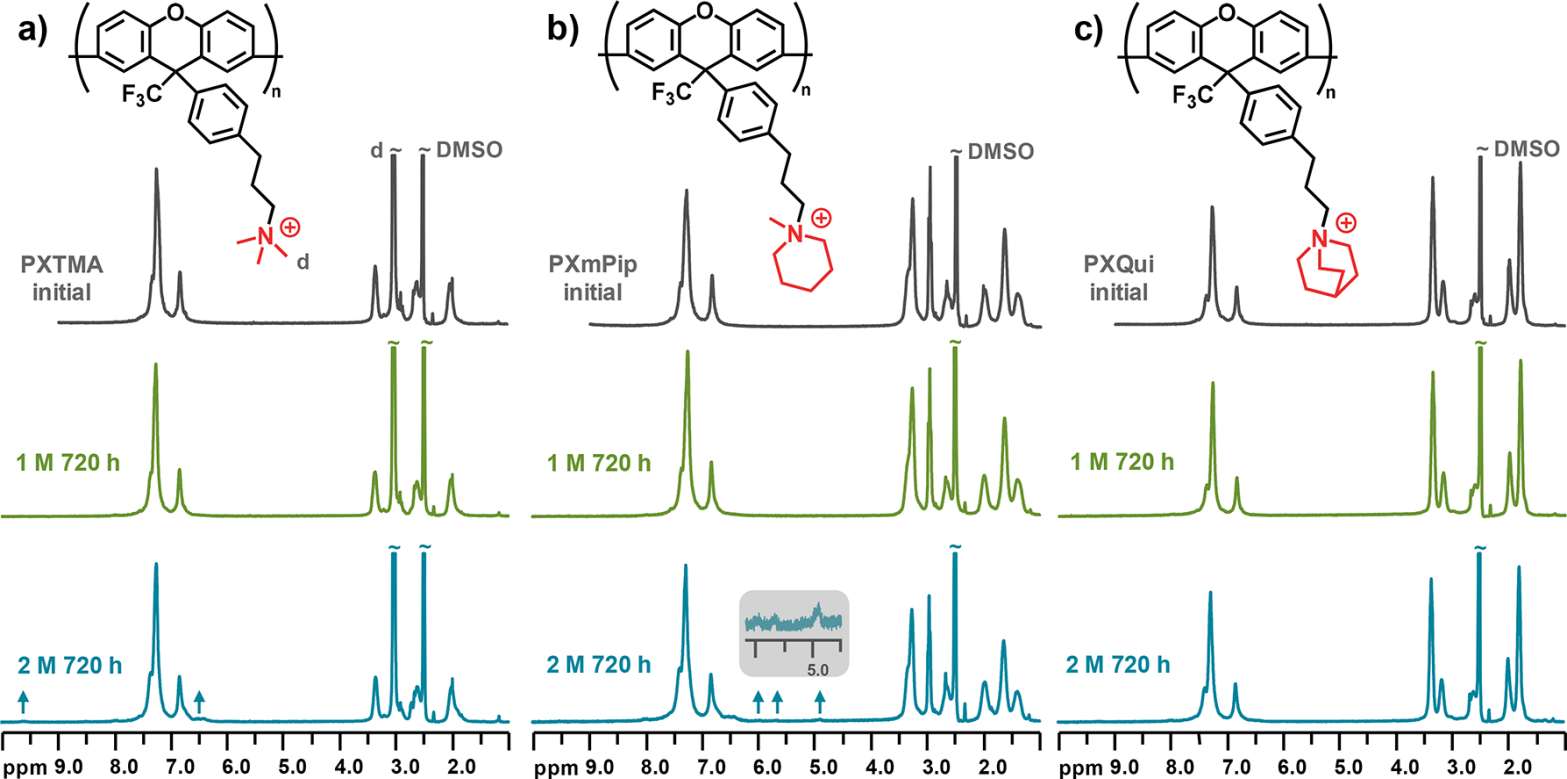
^1^H NMR spectra of (a) PXTMA, (b) PXmPip, and (c) PXQui before and after alkaline treatment during 720 h in 1 M aq. NaOH at 80 °C and 2 M aq. NaOH at 90 °C, respectively.

The level of ionic loss was estimated by comparing the integrals of the new signals to that of the aromatic region assigned to the intact polymer backbone. PXQui was the most stable AEM in this study, with no detectable ionic loss after storage in 2 M aq. NaOH for 720 h at 90 °C. This may be explained by the bulky cage-like structure of the Qui cation, which may provide steric hindrance and conformational constraints to hinder OH^–^ attack, as previously reported by us^26,30^ and others.^31^ Under the same conditions, the ionic loss of PXTMA and PXmPip was estimated to be approximately 3 and 4%, respectively.

Aryl-ether-containing polymers may be susceptible to backbone cleavage by OH^–^ attack.^32,33^ This becomes especially serious if the ether bond is activated by nearby electron-withdrawing groups. To further verify the chemical stability of the PX backbone, TGA analysis was conducted on AEM samples after the alkaline treatments to complement the ^1^H NMR data. The thermal decomposition process, and hence the TGA profile, is generally very sensitive to any change in the ionic content and to the precise molecular structure of a polymer sample. As shown in Figure 4, the TGA traces of all AEMs after treatment in 1 M aq. NaOH at 80 °C for 720 h coincided well with the pristine samples. This not only demonstrated the stability of the xanthene backbone but also confirmed the stability of the tethered QA cations. When the harsher alkaline condition was applied, i.e., 2 M aq. NaOH at 90 °C during 720 h, the magnitude of the first weight loss step decreased notably for samples PXTMA and PXmPip, which was correlated with the loss of QA cations during the alkaline treatment. Still, no sign of the presence of any phenolic groups or backbone degradation was detected after the alkaline treatment. In conclusion, the TGA data were in excellent agreement with the NMR data which demonstrated the high alkaline stability of the present AEMs, both the QA cations and the PX backbone. In the present case, PX has no electron-withdrawing group in close proximity to the backbone ether group to activate C–O bond cleavage.^34^ Similarly, QA-functionalized poly(phenylene oxide)s are typically resistant toward backbone degradation under alkaline treatment, provided that the cations are placed on long alkyl side chains away from the backbone.^35,36^

**Figure 4:**
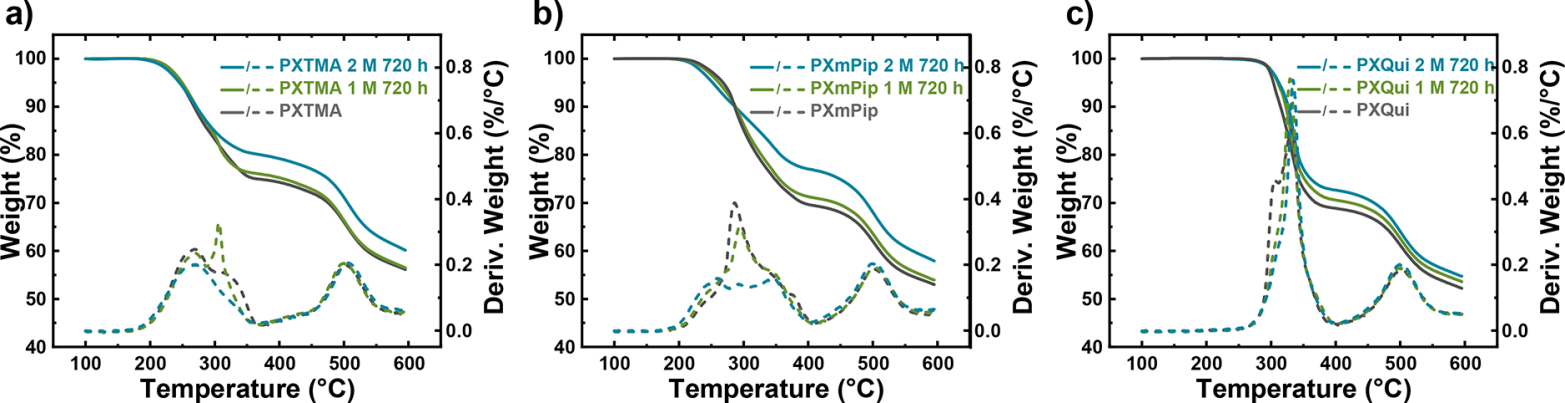
TGA traces and corresponding derivative curves of (a) PXTMA, (b) PXmPip, and (c) PXQui AEMs before and after alkaline treatment during 720 h, recorded in a N_2_ atmosphere at a heating rate of 10 °C min^–1^.

In conclusion, a series of QA-functionalized PXs were prepared through a straightforward synthesis route and studied as a new class of alkali-stable AEMs. The ketone monomer TFAp-Br was readily synthesized in high yield and was found to be highly reactive in polyhydroxyalkylations with biphenol, giving a high molecular weight bromoalkylated PX. After quantitative quaternization reactions, AEMs with a suitable IEC range (2.0–2.3 mequiv g^–1^) were cast from solution, eliminating any need for copolymerization. The membranes showed an attractive combination of high hydroxide conductivity and alkaline stability, and the alkyl-tethered QA cations, especially Qui, were determined to have an excellent stability under harsh alkaline treatment over a long period of time. Special attention was placed on the alkaline stability of the PX backbone, but no sign of any degradation was detected by NMR and TGA analysis. Hence, we found AEMs based on QA-tethered PX to be viable materials for use in AEMFCs and AEMECs, and in the next step the AEMs will be further evaluated in single cells.

## Supporting Information Available

Experimental details, NMR spectra, TGA and DSC data, SAXS profiles, photographs of AEMs, and tables of AEM properties (PDF)
