# Supplementary material for: Alkali-Stable Anion Exchange Membranes Based on Poly(xanthene)
Source: ACS Macro Lett. 2022 Dec 20;12(1):20–5. doi: 10.1021/acsmacrolett.2c00672 (PMC9850910; doi:10.1021/acsmacrolett.2c00672)
Supplement: Supplementary file 1 — mz2c00672_si_001.pdf [file mz2c00672_si_001.pdf]

Supporting information

Alkali-Stable Anion Exchange Membranes Based on Poly(xanthene)

Dong Pan, Si Chen, and Patric Jannasch*

Polymer & Materials Chemistry, Department of Chemistry, Lund University,

P.O. Box 124, SE-221 00, Lund, Sweden

*Email: patric.jannasch@chem.lu.se

Experimental

Materials

1-Bromo-3-phenylpropane (98%, Sigma-Aldrich), anhydrous $AlCl_3$ (ReagentPlus®, 99%, Sigma-Aldrich), trifluoroacetic anhydride (ReagentPlus®, 99%, Sigma-Aldrich), 4,4'-biphenol (97%, Sigma-Aldrich), trifluoromethanesulfonic acid (TFSA, 99%, Sigma-Aldrich), methanol (reagent grade, Sigma-Aldrich), *N*-methyl-2-pyrrolidone (NMP, reagent grade, Sigma-Aldrich), trimethylamine (TMA, 45 wt% in H_2O, Sigma-Aldrich), *N*-methyl-piperidine (98%, Sigma-Aldrich), quinuclidine (97%, Sigma-Aldrich), DMSO-d_6 (99.96 at% D, Sigma-Aldrich), chloroform-*d* (99.8 at% D, Sigma-Aldrich), NaBr (99%, VWR), $NaNO_3$ (99%, Sigma-Aldrich), Na_2SO_4 (anhydrous, >99%, Sigma-Aldrich), $AgNO_3$ (99%, Sigma-Aldrich), NaOH (99%, pellets, VWR), dimethylacetamide (DMAc, reagent grade, Sigma-Aldrich), diethyl ether (reagent grade, VWR) and ethyl acetate (reagent grade, Sigma-Aldrich) were used as received. Dichloromethane (DCM) was dried using MBraun dry solvent dispenser system MB-SPS 800.

Monomer synthesis

1-Bromo-3-(trifluoroacetylphenyl)-propane (TFAp-Br) was synthesized in one step using a modified procedure.[1] A 250 mL Schlenk flask was equipped with a magnetic stirrer and charged with $AlCl_3$ (0.09 mol, 12 g), before sealed with a rubber septum. The flask was evacuated and backfilled with N_2 three times. Dry dichloromethane (DCM, 120 mL) was added and the flask was placed in an ice batch. Then, trifluoroacetic anhydride (11.82 g, 0.056 mol) were added slowly. 1-Bromo-3-phenylpropane (8 g, 0.04 mol) was dissolved in 40 mL DCM and the solution was dropwise added to the Schlenk flask. Subsequently, the reaction mixture was stirred at room temperature for 2 h. A color change of the mixture from yellow to purple was observed during the reaction. The mixture was then poured into water and extracted with ethyl acetate three times. The organic phase was collected and dried with Na_2SO_4 before evaporating the solvent. The acquired brown liquid was distilled at 160 °C under vacuum, giving 10.87 g TFAp-Br (92% yield) as a colorless liquid.

Polycondensation

Precursor polymer PX-Br was synthesized in a polycondensation reaction between monomer TFAp-Br and 4,4'-biphenol. 4,4'-Biphenol (3 g, 10.2 mmol), TFAp-Br (1.89 g, 10.2 mmol) and DCM (14 mL) was charged to a 25 mL round-bottom flask in an ice bath and stirred until homogeneous. Trifluoromethanesulfonic acid (TFSA, 2 mL, 22.4 mmol) was then dropwise added to the mixture, and the polymerization was allowed to proceed at room temperature for 2 h. The resulting dark, viscous mixture was diluted with DCM, before precipitating the product in methanol to obtain light-yellow fibers. The precipitate was further washed in methanol at 50 °C, then dissolved again in DCM and re-precipitated in methanol. Finally, the product PX-Br was dried under vacuum at room temperature.

Menshutkin reactions on PX-Br were performed using procedures adapted from our previous work.[2] PX-Br (0.45 g, 1 eq.) was fully dissolved in 20 mL dimethylacetamide (DMAc) before adding trimethylamine (TMA, 1.6 ml, 45 wt% in H_2O solution, 10 eq.). The reaction was kept at room temperature for 4 days, before slowly pouring the dark-orange solution into diethyl ether. The fibrous precipitate was washed in fresh diethyl ether, before collected and dried to quantitatively give PXTMA as a pinkish powder. PXmPip

and PXQui were prepared using a similar procedure, however using 10 and 2 eq. of each amine, respectively, and conducting the reactions at 85 °C for 7 days.

Membrane preparation

First, 5 wt% polymer solutions in dimethyl sulfoxide (DMSO) were prepared and passed through a PTFE filter (ϕ = 5 μm). The solutions were poured onto a petri dish (ϕ = 5 cm) and AEMs were cast during 2 days at 80 °C in an air-circulating oven. Transparent and uniform membranes were obtained for all the polymers. The AEMs were subsequently immersed in aq. NaBr for 2 days, then washed and stored in deionized water (DI water).

Structural characterization

^{1}H, ^{13}C and ^{19}F NMR spectra of all the compounds and polymers were recorded with Bruker DRX 400 spectrometer at 400, 101 and 376 MHz, respectively, using either $CDCl_3$ (δ = 7.26 ppm) or DMSO-d_6 (δ = 2.50 ppm) as solvent. In some cases, trifluoroacetic acid (TFA) was added to the sample to shift the broad water signal in order to reveal signals otherwise overlapped.

Size exclusion chromatography (SEC) was used to determine the molecular weight (M_n), dispersity (*Đ*) of PX-Br. The Malvern Viscotek instrument was equipped with two PL-Gel Mix-B LS columns (2 × 30 cm) and OmniSEC triple detectors (refractive index, viscosity and light scattering). Chloroform was used as ecluent at 35 °C at a flow rate of 1 mL min^{-1}. Calibration was carried out using a standard polystyrene (M_n = 96 kDa, *Đ* = 1.03, polymer Laboratories Ltd.). PX-Br was dissolved 24 h prior to the measurement and passed through a PTFE filter before analysis (pore size 0.2 μm).

Small angle X-ray scattering (SAXS)

The morphology of the AEMs were studied by small angle X-ray scattering (SAXS). AEM samples in the Br^- form were prepared and dried prior to the measurements. The experiments were carried out on a SAXLAB instrument (JJ X-ray Systems ApS, Denmark) equipped with a Pilatus detector. Data was collected in the q-range 0.14–0.8 nm^{-1}.

Ion exchange capacity, water uptake and swelling ratio

The ion exchange capacity (IEC) of the AEMs was determined by Mohr titrations. Approximately 0.03 g of each AEM sample in the Br^- form was dried under vacuum at 50 °C for 48 h, before precisely weighed, and submerged in 25 mL 0.2 M aq. $NaNO_3$ for 48 h. The resulting solution was titrated in portions (5 mL × 4 times) with 0.01 M aq. $AgNO_3$ using K_2CrO_4 as color indicator. Taking the average endpoint of the 4 titrations, the IEC was calculated using the equation:

$$IEC_{OH} = \frac{IEC_{Br}}{1 - 0.0629 \times IEC_{Br}} \quad (1).$$

The water uptake (WU) of the AEMs in the OH^- form was measured gravimetrically. Dry samples in the Br^- form were weighed ($W_{dry, Br}$) and ion-exchanged in 1 M aq. NaOH for 48 h, before repeatedly washed and stored in freshly degassed DI water at 20, 40, 60 and 80 °C, respectively. At 20 °C, the AEMs were

equilibrated for 24 h, while at 40, 60 and 80 °C the equilibration was done during 8 h. Subsequently, the sample was taken out, quickly wiped with tissue paper and weighed ($W_{wet, OH}$). Then the water uptake was calculated as follows, with $W_{dry, OH}$ acquired using titrated IEC_{Br}:

$$W_{dry,OH} = W_{dry,Br} \times (1 - 0.0629 \times IEC_{Br}) \quad (2),$$

$$WU = \frac{W_{wet,OH} - W_{dry,OH}}{W_{dry,OH}} \times 100\% \quad (3).$$

Dimensional changes in length (l) and thickness (t) of the AEMs were recorded in parallel with the WU measurements to investigate the in-plane (SW_i) and through-plane (SW_{th}) swelling of the AEMs. Swelling ratios were then calculated as:

$$SW_{\mathrm{i}} = \frac{l_{\mathrm{wet}} - l_{\mathrm{dry}}}{l_{\mathrm{dry}}} \times 100\% \quad (4),$$

$$SW_{\mathrm{th}} = \frac{t_{\mathrm{wet}} - t_{\mathrm{dry}}}{t_{\mathrm{dry}}} \times 100\% \quad (5).$$

Conductivity measurements

Electrochemical impedance spectroscopy (EIS) was applied to measure the OH^- conductivity of the samples immersed in air-free DI water between 20−80 °C. Sample AEMs were cut into a square shape (1.4 × 1.4 cm) and immersed in 1 M aq. NaOH at room temperature for 48 h. During this time, the alkaline solution was replaced 3 times with fresh ones to ensure complete ion exchange. Next, the samples were thoroughly washed with air-free DI water and stored in a N_2 ventilated desiccator prior to measurements. The measurements were conducted using a 2-probe cell mounted in a Novocontrol high resolution dielectric analyzer V 1.01S operating at 10 mV between 10^7−10^0 Hz. For each AEM, two samples were measured and the average conductivity value was reported.

Thermal characterizations

The thermal decomposition of PX-Br and the AEM polymers in the Br^- form was studied by thermogravimetric analysis (TGA). TGA traces were recorded with a TA Instruments TGA Q500. During the experiment, the samples were preheated to 150 °C and equilibrated for 20 min before heating from 50 to 600 °C at a rate of 10 °C min^{-1} under N_2 atmosphere. The temperature at 95% weight loss was reported as the decomposition temperature ($T_{d,95}$).

Differential scanning calorimetry (DSC) was performed on PX-Br using a TA Instruments DSC Q2000. Approximately 3 mg PX-Br was first heated 300 °C at a rate of 10 °C min^{-1}, cooled to 25 °C, then heated to 300 °C again at the same heating rate.

Alkaline stability

AEM samples were placed in sealed pressure-resistant tubes containing 1 M and 2 M aq. NaOH, which were then kept at 80 °C and 90 °C, respectively. After predetermined storage times (168, 336 and 720 h),

samples were taken out and ion-exchanged in aq. NaBr for 48 h. ^{1}H NMR spectroscopy was then performed on dried samples using DMSO-d_6 as solvent with 5-10 vol% TFA added. In addition, the same samples were analyzed by TGA using the procedures described above.

Figures and Tables

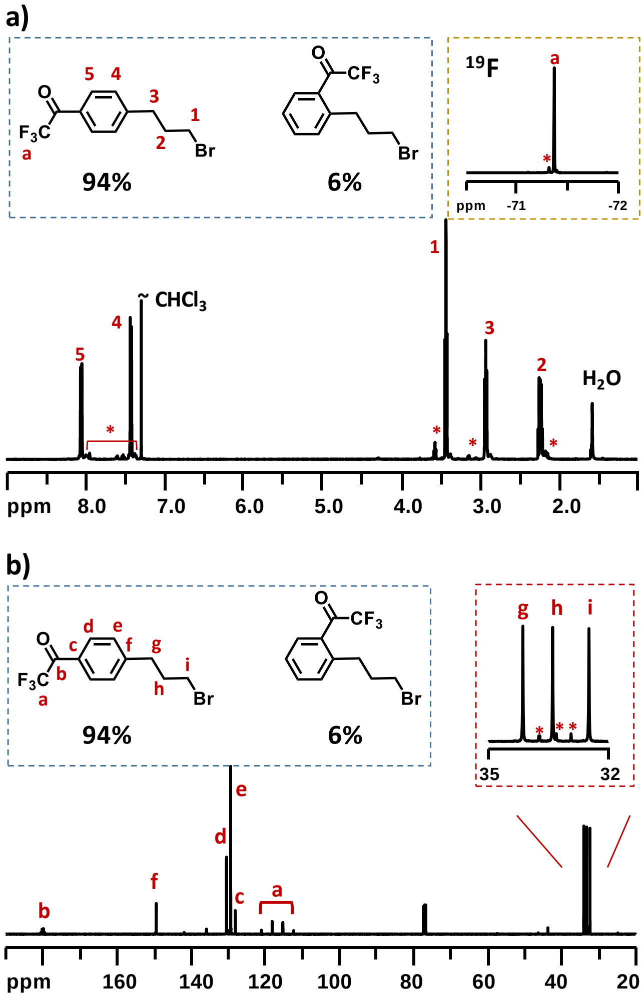

Figure S1. (a) ^{1}H, ^{19}F (in the dashed box) and (b) ^{13}C NMR spectra of monomer TFAp-Br in $CDCl_3$. Signals marked by asterisks arise from the ortho-substituted isomer.

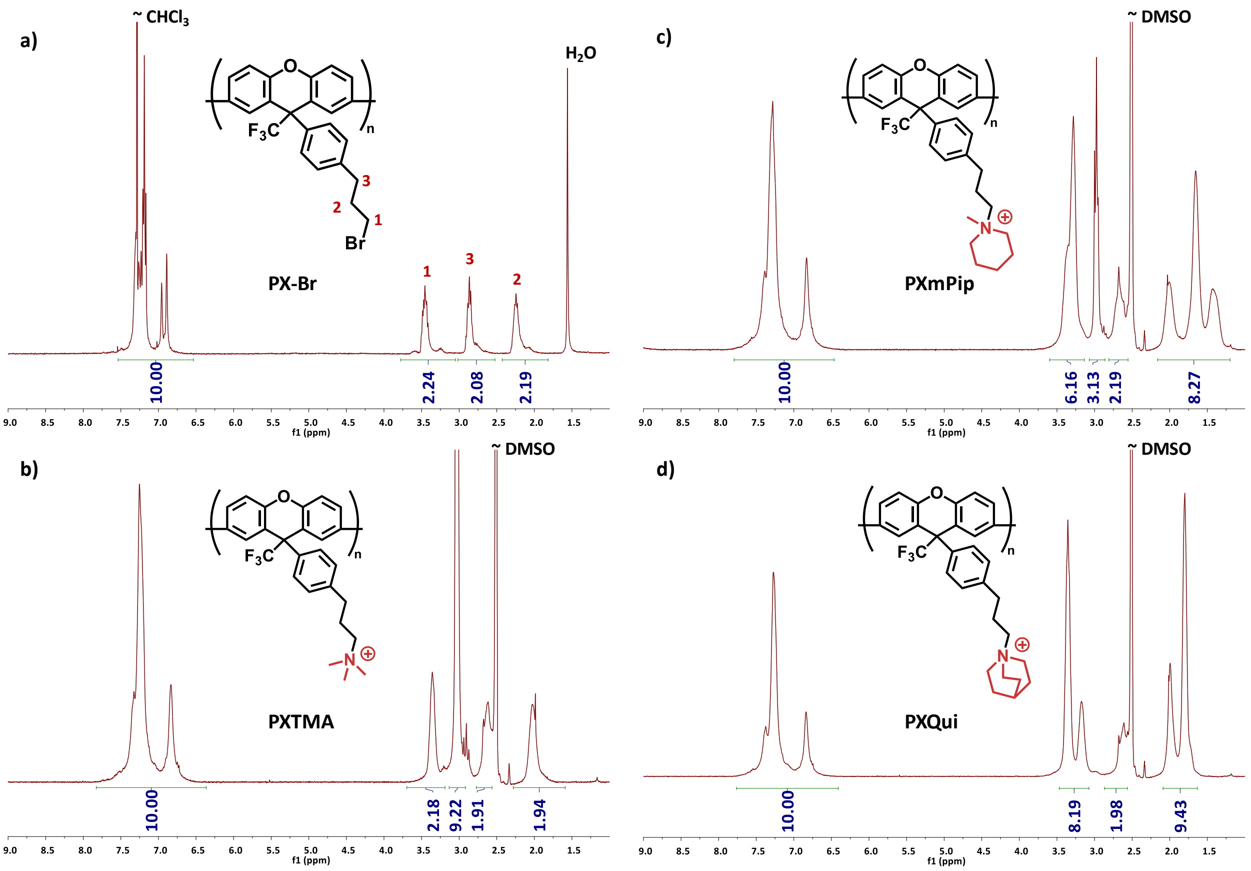

Figure S2. ^{1}H NMR spectra of (a) precursor polymer PX-Br in $CDCl_3$, (b-d) AEM polymers in DMSO-d_6 with TFA.

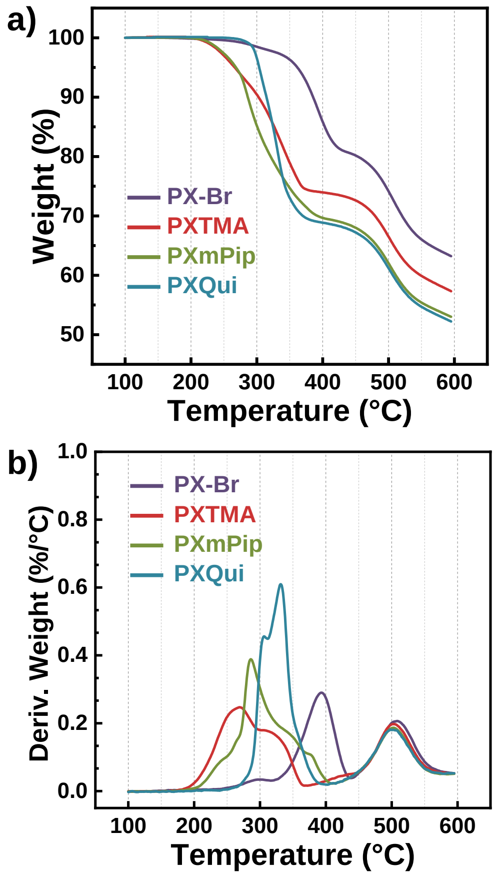

Figure S3. (a) TGA traces and (b) corresponding first derivatives of PX-Br and AEM samples (in the Br^- form) recorded under N_2 atmosphere at a heating rate of 10 °C min^{-1}.

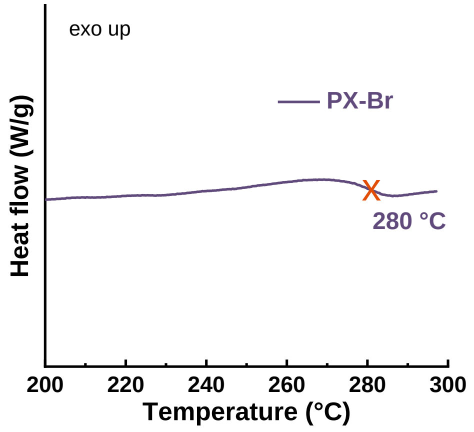

Figure S4. DSC curve recorded during the second heating cycle of precursor polymer PX-Br at a heating rate of 10 °C min^{-1}.

Table S1. The properties of the AEMs.

AEM	IEC_{OH} (mequiv.g^{-1})		WU_{20}[c]	WU_{80}[c]	SW_{i20}[c]	SW_{i80}[c]	SW_{th20}[c]	SW_{th80}[c]	σ_{20}[c]	σ_{80}[c]	λ_{80}[c]
	by NMR[a]	by titration[b]	(%)	(%)	(%)	(%)	(%)	(%)	(mS cm^{-1})	(mS cm^{-1})	
PXTMA	2.26	2.27	92	175	24	38	28	54	60	129	43
PXmPip	2.08	2.05	75	149	24	37	14	32	53	114	40
PXQui	2.03	2.03	85	149	21	34	11	32	49	104	41

[a] Theoretical value determined by the chemical structure and ^{1}H NMR spectroscopy. [b] Experimental value obtained by Mohr titrations. [c] Measured on AEMs in the OH^- form in the fully hydrated state.

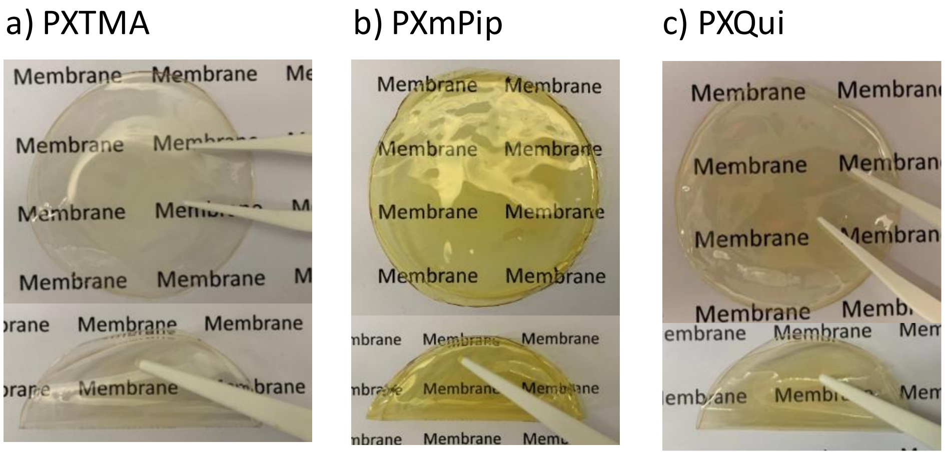

Figure S5. Photographs of the AEMs.

Table S2. Properties of PX-Br and the AEMs.

Sample	M_n (kDa)	$Đ$	$T_{d, 95}$ (°C)
PX-Br	117	4.1	361
PXTMA	-	-	247
PXmPip	-	-	268
PXQui	-	-	303

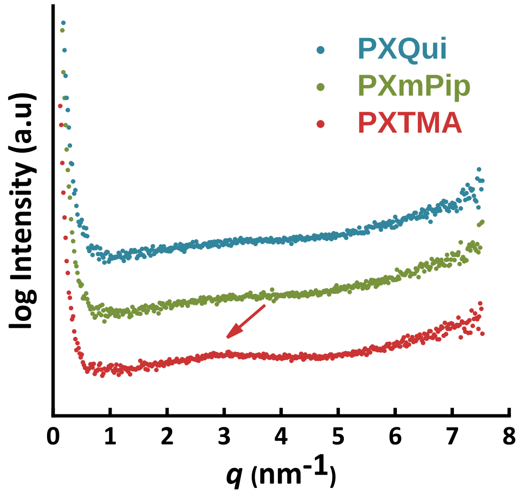

Figure S6. SAXS profiles of dry AEMs in the Br^- form.

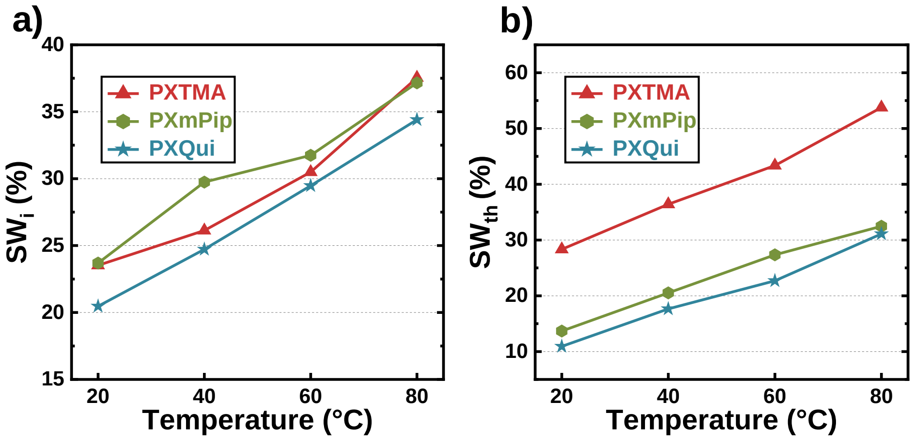

Figure S7. (a) In-plane and (b) through-plane swelling ratios of the AEMs in the OH^- form, measured between 20 and 80 °C.

Table S3. OH^- conductivity of PXTMA compared with the data of recently reported AEMs based on various polymer backbones carrying TMA cations.

AEM	IEC_{OH}[a] (mequiv g^{-1})	WU[b] (%)	λ[c]	σ[b] (mS cm^{-1})	Backbone type	Cation	Ref.
PXTMA	2.27	175	43	129	Poly(xanthene)	TMA	This work
$PBPA^+$	2.70	145	30	122	Poly(biphenyl alkylene)	TMA	3
p-TPN1	2.16	65	17	81	Poly(*p*-terphenyl alkylene)	TMA	4
m-TPN1	2.18	70	18	112	Poly(*m*-terphenyl alkylene)	TMA	4
P(4PA-co-2PA)-24	2.17	-	~11	~90	Poly(quaterphenyl alkylene)	TMA	5
FLN-55	2.50	180	40	120	Poly(fluorene alkylene)	TMA	6

[a] IEC value in the OH^- form determined by Mohr titrations. [b] Data acquired at 80 °C. [c] Hydration number in the OH^- form at 80 °C.

References

(1) Marestin, C.; Chatti, S.; Mercier, R. Synthesis of poly(aryl ether)s bearing phosphonated side-chains from phosphonate ester-containing bisphenols. *Polymer* **2021,** *222*, 123647.

(2) Dang, H. S.; Jannasch, P. A comparative study of anion-exchange membranes tethered with different hetero-cycloaliphatic quaternary ammonium hydroxides. *J. Mater. Chem. A* **2017,** *5* (41), 21965-21978.

(3) Lee, W. H.; Kim, Y. S.; Bae, C. Robust Hydroxide Ion Conducting Poly(biphenyl alkylene)s for Alkaline Fuel Cell Membranes. *ACS Macro Lett.* **2015,** *4* (8), 814-818.

(4) Lee, W. H.; Park, E. J.; Han, J.; Shin, D. W.; Kim, Y. S.; Bae, C. Poly(terphenylene) Anion Exchange Membranes: The Effect of Backbone Structure on Morphology and Membrane Property. *ACS Macro Lett.* **2017,** *6* (5), 566-570.

(5) Jiang, T.; Wu, C.; Zhou, Y.; Cheng, S.; Yang, S.; Wei, H.; Ding, Y.; Wu, Y. Highly stable poly(p-quaterphenylene alkylene)-based anion exchange membranes. *J. Membr. Sci.* **2022,** *647*, 120342.

(6) Maurya, S.; Noh, S.; Matanovic, I.; Park, E. J.; Villarrubia, C. N.; Martinez, U.; Han, J.; Bae, C.; Kim, Y. S. Rational design of polyaromatic ionomers for alkaline membrane fuel cells with > 1 W cm(-2) power density. *Energy Environ. Sci* **2018,** *11* (11), 3283-3291.
